# Predicting anthropometric body composition variables using 3D optical imaging and machine learning

**DOI:** 10.3389/fbinf.2026.1722578

**Published:** 2026-05-13

**Authors:** Gyaneshwar Agrahari, Kiran Bist, Monika Pandey, Jacob Kapita, Zachary James, Jackson Knox, Steven Heymsfield, Sophia Ramirez, Peter Wolenski, Nadejda Drenska

**Affiliations:** 1 Department of Mathematics, Louisiana State University, Baton Rouge, LA, United States; 2 Pennington Biomedical Research Center, Baton Rouge, LA, United States

**Keywords:** 3D imaging, body composition, p-Laplacian-regression, semi-supervised learning, support vector regression (SVR)

## Abstract

Accurate prediction of anthropometric body composition variables, such as Appendicular Lean Mass (ALM), Body Fat Percentage (BFP), and Bone Mineral Density (BMD), is essential for early diagnosis of several chronic diseases. Currently, researchers rely on Dual-Energy X-ray Absorptiometry (DXA) scans to measure these metrics; however, DXA scans are costly and time-consuming. This work proposes an alternative to DXA scans by applying statistical and machine learning models on biomarkers (height, volume, left calf circumference, etc.) obtained from 3D optical images. The dataset consists of 847 patients and was sourced from the Pennington Biomedical Research Center. Extracting patients’ data in healthcare faces many technical challenges and legal restrictions. However, most supervised machine learning algorithms are inherently data-intensive, requiring a large amount of training data. To address this challenge, we compare the standard supervised to a semi-supervised p-Laplacian model, which leverages the limited data by incorporating the unlabeled patient information. To our knowledge, this paper is the first to demonstrate the application of a game-theoretic p -Laplacian model for regression in healthcare. Our p -Laplacian model yielded errors of ∼13% for ALM, ∼10% for BMD, and ∼20% for BFP when the training data accounted for 10 percent of all data. Among the supervised algorithms we implemented, Support Vector Regression (SVR) performed the best for ALM and BMD, yielding errors of ∼8% for both, whereas Least Squares SVR performed the best for BFP with ∼11% error when trained on 80% the data. Our findings position the p -Laplacian model as a promising tool for healthcare applications, particularly in a data-constrained environment with limited labeled data.

## 1 Introduction

With the rise of applications of artificial intelligence to healthcare, personalized treatment and medicine have seen tremendous advances, Bohr and Memarzadeh (2020). Estimating biometrics accurately is essential to gaining insights into personalized human health. Health researchers and nutritionists use several types of biometrics - metabolic (cholesterol, insulin),S performance-based (speed, balance) and physiological (lean mass, body fat). ALM, BFP, and BMD are three significant physiological biometrics. ALM is linked to malnutrition risk and age-related frailty, and serves as a risk factor for adverse treatment outcomes associated with metabolic syndrome (MetS) and other clinical conditions, Bennett et al. (2024). BFP is associated with obesity and cardiovascular diseases, Zeng et al. (2012). BMD is instrumental in determining osteoporosis conditions, Fathima et al. (2022).

Researchers have been using Dual-energy X-ray Absorptiometry, or DXA, to estimate these biometrics directly. Using a scan like DXA, however, is costly, time-consuming, and inconvenient for both researchers and patients. An alternative approach is to estimate the biometrics by applying mathematical models to body measurements like height, weight, leg volume, and waist circumference. This approach involves two key steps: (1) the extraction of the body measurements and (2) application of mathematical models on these measurements. There have been developments in these two key steps. Recent advancements now enable us to capture 2D and 3D digital images of people not only at research labs and hospitals but also at home using smartphone applications (McCarthy et al., 2023). The body measurements can be extracted from these images using a computer program. Such advancements have made the extraction of the body measurements cost-effective and time-efficient. For the second key step, traditionally, statistical tools like anthropometric body composition prediction equations have been used to make the estimations, which typically do not account for non-linear relationships (Marin-Jimenez et al., 2022). In the past decade, however, machine learning algorithms have been proven to be more accurate and more efficient in estimating biometrics (Higgins et al., 2021). Models, especially supervised algorithms, require a substantial amount of training data (Adadi, 2021). The main consequence of relying on DXA is that the measurements are difficult to obtain, which limits the amount of labeled data available for training predictive models in clinical settings.

This raises a central question: can accurate prediction of ALM, BMD, and BFP be achieved when only a limited amount of DXA-labeled data is available? In recent years, several machine learning algorithms have been developed that require fewer data to be trained without significant loss of accuracy (Wang et al., 2020). Specifically, semi-supervised algorithms, such as semi-supervised support vector machines and graph-based label propagation have been proven efficient when training data is scarce (Van Engelen and Hoos, 2019). More recently, researchers have begun using semi-supervised algorithms in many bio-informatics applications.

In this paper, we will focus on such a semi-supervised algorithm - p -Laplacian based regression. We will use it alongside several supervised learning algorithms to predict the biometrics: ALM, BFP, and BMD. These models are selected to represent different training strategies under varying levels of labeled data availability. This enables a direct comparison between the performance of supervised and semi-supervised approaches. Our results highlight the p -Laplacian model as a strong candidate for healthcare applications, especially when data are limited.

This paper is structured as follows. In Section 2, we highlight the existing literature on predicting ALM, BFP and BMD using statistical and machine learning algorithms. In Section 3, we describe how we obtained anthropometric measurements and tested the models. Section 4 includes a descriptive summary of the data. Section 5 describes the supervised algorithms we implemented, and in Section 6 we describe the only semi-supervised algorithm - p -Laplacian based regression - in detail. Section 7 encompasses all the main results from our analysis. We discuss our main findings and future work, as well as some limitations of our work, in Section 8.

## 2 Literature review

Previous studies such as Wu et al. (2021); Shioji et al. (2017); Alves et al. (2021); Marazzato et al. (2024) have shown that machine learning models are accurate in predicting ALM, BMD, and BFP. Wu et al. (2021) used random forest, gradient boosting, neural network, and linear regression models to predict BMD. They found that the gradient boosting model performed the best on a dataset comprised of 1103 individual Single Nucleotide Polymorphisms (SNPs). However, when they used Genetic Risk Scores (GRS’s) as input data, all models performed similarly to one another.

Shioji et al. (2017) used a dataset of 135 Japanese women of ages over 50 to train and predict BMD and bone loss rates using artificial neural networks. The authors discovered that neural networks performed better than multiple regression approaches.

Alves et al. (2021), discovered that the sex of a person influences the prediction of BFP. They used a dataset composed of both male (84) and female (79) subjects and developed various machine learning models to predict BFP on the combined dataset as well as on the datasets split up by sex. The model types used to predict BFP were random forest regression, extreme gradient boosting, decision tree, support vector regression, multi-layer perceptron (MLP) regression, and least square support vector regression (LSSVR). They observed that when using the combined dataset, the mean absolute error (MAE) range was between 3.569 and 9.859; the LSSVR achieved the best performance, and MLP reached the worst when using the combined dataset. However, the error range decreased to a low of 2.756 with LSSVR and a high of 6.15 with MLP when the dataset was restricted to males only. For the females, the error ranged from 4.004 with LSSVR to 8.003 with MLP.

Marazzato et al. (2024) used a neural network with 1 input, 3 hidden, 2 dropout, and 1 output layers to predict ALM from 10 demographic and 43 digital anthropometric measurements acquired using a 3D optical scanner. The used a dataset with 576 subjects. The results, showing small mean, absolute, and root-mean square errors, helped Marazzato et al. to recommend the use of neural networks to predict ALM.

Barbosa-Silva et al. (2021) used a dataset of 190 participants (118 women and 72 men) to predict ALM using muscle thickness measurements extracted via ultrasound. They recommended using two prediction equations after observing that the developed equations produced unbiased ALM estimates that were close to the reference measurements found by the Ultrasound scanner.

There have been limited applications of semi-supervised (SSL) algorithms in predicting ALM, BMD, and BFP. Zheng et al. (2021) used a semi-supervised learning self-training algorithm to predict BMD from plain hip X-ray images, and achieved a high Pearson correlation coefficient of 0.8805. Our work initiates the application of SSL algorithms in the prediction of ALM and and BFP. To our knowledge, this technique has not been used to predict ALM or BFP in previous research. Also, this is the first investigation of the use of SSL to predict BMD without using data obtained from X-ray images in prediction. Table 1 summarizes the literature review on the models used to predict ALM., BFP, and BMD.

**TABLE 1:** Concise summary of related ML/SSL studies for ALM/BFP/BMD prediction.

Study	Target	Inputs/Modality	N	Key result (as reported)
Wu et al. (2021)	BMD	Genomics (SNP/GRS) + phenotype	1103	Gradient boosting best; models similar when using GRS
Shioji et al. (2017)	BMD	Clinical variables (postmenopausal cohort)	135	ANN outperformed multiple regression
Alves et al. (2021)	BFP	Anthropometrics	163	MAE: 3.57–9.86 (combined); 4.00–8.00 (female)
Marazzato et al. (2024)	ALM	3D optical anthropometrics + demographics	576	NN reported low error (MAE/RMSE)
Barbosa-Silva et al. (2021)	ALM	Ultrasound muscle thickness	190	Regression equations yielded unbiased estimates
Zheng et al. (2021)	BMD	Hip X-ray images (SSL)	819	Pearson r=0.8805
Feng et al. (2026)	LM,BF, and BMC	3D human body polygonal meshes	287	Deep regression with 90–10 training-validation split

Recent work has explored end-to-end deep learning approaches for predicting body composition from 3D body scans. For instance, Feng et al. (2026) proposed a voxel-based deep regression framework that integrates 3D voxel representations with demographic features. Their study was conducted on a dataset of 287 adult participants, in 90–10 training-validation splits, and predicts Lean Mass (LM), Body Fat (BF), and Bone Mineral Content (BMC). Their model achieves RMSE values of 0.0392 kg, 0.0305 kg, and 0.0862 g for lean mass, fat mass, and bone mineral content, respectively.

## 3 Methodology

### 3.1 Data extraction

All the body measurements, here referred to as biomarkers, were gathered at the Pennigton Biomedical Research Center (PBRC) facility (Sobhiyeh et al., 2021). There were 847 participants, each of whom gave informed consent to be a part of the study. The data used in this study was obtained from the National Institutes of Health–funded *Shape Up! Adults* study (NIH R01 DK109008, R01 DK111698), which is registered at *clinicaltrials.gov* (NCT03637855).

Each participant had measurements of biomarkers such as the circumference and length of chest, waist, hip, arm, thigh, etc., deduced from the 3D image. The image was created from 2D images taken by one of the three different machines, namely (i.) the Proscanner, which uses three stationary cameras aligned vertically on a column to image a person standing on a rotating turntable, (ii.) the Styku machine, which has a similar design as the Proscanner but with a single camera, and finally (iii.) the SS20, which has five cameras positioned on four different vertical columns to capture the image (Sobhiyeh et al., 2021).

**FIGURE 1:**
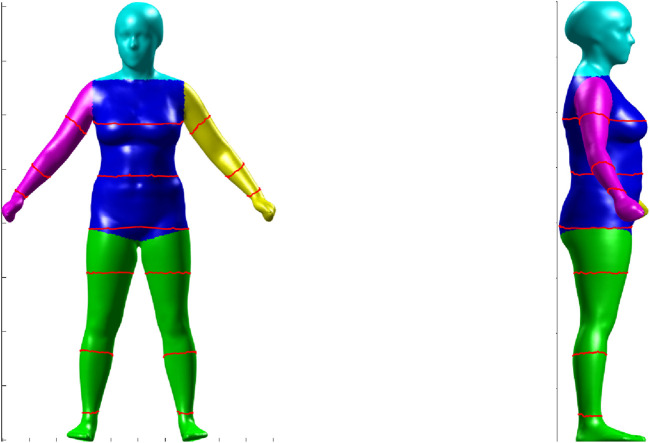
Segmented 3D human body model shown from front (left) and side (right) views. Colors indicate anatomical regions, and red contours denote circumferential measurement locations used for anthropometric feature extraction.

The images from the machines were used to create a 3D triangular mesh with the 3D vertices representing cloud points and the area between them making a surface. Then, software created in Matlab by Math Consultation Clinic $\left(MC^{2}\right)$ group at Louisiana State University corrects any errors in the meshing and fills any holes in the mesh to produce a three-dimensional avatar, as shown in Figure 1 (Sobhiyeh et al., 2021).

After the mesh is corrected, it is segmented into various parts of the body such as the center, right arm, right leg, left arm, and left leg, as in Figure 1. Then, the biomarkers for the patient are computed from the segmented mesh (Sobhiyeh et al., 2021). Out of all the measured biomarkers, we have used 44 biomarkers in this study.

The data collected involved 847 patients. However, 332 of the 847 patients had incomplete biomarker information due to measurement collection difficulties. Since these 332 patients have at least one biomarker missing, we cannot run the algorithms on them, and therefore we had to disregard these patients’ data from the data set we use. This has left us with 515 patients, all with complete data. Imputation was not performed. Participants with missing biomarkers were excluded to avoid introducing additional uncertainty into predictive models. Race variables were excluded due to the limited and unbalanced size of racial groups, which could lead to unreliable estimates; See Section 8.1 for a further detailed discussion. Since sex is a crucial categorical variable in determining ALM, BMD, and BFP, we decided to break down the data sets, ending up with three - males, females, and combined. The results presented in this paper are for these three groups.

### 3.2 Model testing

We tested all the models on three datasets - Male, Female, and Combined - for each target variable: ALM, BMD, and BFP. We investigated the effects of normalizing the data using both the StandardScaler() and MinMaxScaler() classes from scikit-learn. The StandardScaler() outperformed MinMaxScaler() in our experiments, so we use the StandardScaler() throughout this article. To validate the models’ analysis, we used K -Fold Cross Validation. For the semi-supervised model, we further modified the K -Fold cross validation to train the model on a smaller percentage of training data. In every case, we independently ran the models 10 times where in each run the data was split into K folds randomly.

To ensure that there was no data leakage, we made use of different regularization methods to mitigate overfitting in the presence of correlated predictors across all the models. For the MLP and polynomial regression, we apply L2 regularization. The MLP model used 20% dropout, together with feature scaling performed strictly within each cross-validation fold using fit.transform() on the training data and transform() on the test data. This procedure, combined with regularization, prevents information leakage across folds. For SVR and LS-SVR, margin-based regularization is enforced through the cost parameter. The p-Laplacian model incorporates an explicit graph-smoothness penalty that regularizes predictions over a patient similarity graph, thereby controlling model complexity and mitigating overfitting.

#### 3.2.1 Error analysis

We used Root Mean Square Error (RMSE) in percent, given by Equation 1, to optimize our models. Our goal is to predict ALM, BFP, and BMD with high accuracy represented by lowest RMSE. The RMSE is expressed as a percentage for better comparison across models, target variables, and datasets.

$$\text{RMSE}=\frac{\sqrt{\frac{1}{N}\sum_{i=1}^{N}{\left({\hat{y}}_{i}-y_{i}\right)}^{2}}}{\sqrt{\frac{1}{N}\sum_{i=1}^{N}y_{i}^{2}}}\times 100$$ (1)

where.

$y_{i}=$ True value of ALM, BFP, or BMD,

${\hat{y}}_{i}=$ Predicted value of ALM, BFP, or BMD, and

N= Number of patients in the testing set.

Each model was implemented 10 times where each run was implemented with K -cross validation. The average RMSE for the model was calculated by the taking average over all runs and K -folds.

## 4 Data description

The Pennington Biomedical Research Center in Baton Rouge, Louisiana provided the data used for this research. The collected data was utilized to predict ALM, BMD, and BFP. Additionally, influenced by the dependence of the three target values on sex, we conducted an in-depth data analysis by dividing it into three datasets: Male, Female, and Combined (the entire dataset). Table 2 includes the list of the biomarkers in the dataset.

**TABLE 2:** List of the biomarkers.

Biomarker
Height (cm)
Weight (kg)
Abdomen circumference (cm)
Ankle circumference left (cm)
Arm length left (cm)
Arm volume left (cm^3^)
Bicep circumference left (cm)
Calf circumference left (cm)
Chest (cm)
Collar circumference (cm)
Forearm circumference left (cm)
Head circumference (cm)
Hip circumference (cm)
Horizontal waist (cm)
Inseam left (cm)
Leg volume left (cm^3^)
MidThigh circumference left (cm)
Narrow waist (cm)
Outside leg length left (cm)
Seat circumference (cm)
Surface area arm left (cm^2^)
Surface area leg left (cm^2^)
Surface area torso (cm^2^)
Surface area total (cm^2^)
Thigh circumference left (cm)
Torso volume (cm^3^)
Upper arm circumference left (cm)
Volume (cm^3^)
Waist circumference (cm)
Ankle circumference right (cm)
Arm length right (cm)
Arm volume right (cm^3^)
Bicep circumference right (cm)
Calf circumference right (cm)
Forearm circumference right (cm)
Inseam right (cm)
Leg volume right (cm^3^)
MidThigh circumference right (cm)
Outside leg length right (cm)
Surface area arm right (cm^2^)
Surface area leg right (cm^2^)
Thigh circumference right (cm)
Upper arm circumference right (cm)
Age (years)

Using a correlation matrix, we assessed the relationship between the biomarkers and the target variables (ALM, BMD, and BFP) and identified the top 10 biomarkers for each dataset. See Tables 4–6.

### 4.1 Data cleaning

We removed participants with missing values in order to clean the data. After eliminating the rows with missing entries, we used the remaining data for the training and testing purposes. The final cleaned data set consists of 515 people, their 44 biomarkers extracted from the process described in Section 3.1, and their ALM, BFP, and BMD values obtained from DXA scans. Table 2 lists the biomarkers in the dataset. During data cleaning, we excluded several columns, including “Site”, “Race”, and “Sex”. The Sex variable was removed because the dataset was explicitly partitioned into male, female, and combined cohorts prior to model training.

### 4.2 Descriptive statistics

The dataset consists of 245 males and 270 females. In the combined dataset, the top ten biomarkers demonstrate a strong positive correlation with ALM (over 0.9), a moderate correlation with BMD (0.7–0.81), and a lower correlation with BFP (0.4–0.6). When analyzing the data separately, most of the biomarkers showed a stronger correlation with ALM and BMD in males than in females. However, among the top ten biomarkers, Surface Area Total shows the highest correlation with ALM in both sexes.

For BFP, the correlation with the top ten biomarkers is slightly higher in females (0.6–0.76). In both sexes, Horizontal Waist showed the strongest correlation with BFP. This high correlation is consistent with the research finding that adult women generally have higher body fat percentages than men (Jackson et al., 2002).

For BMD, Surface Area Arm is the most strongly correlated in males, whereas Height is the most influential factor in females. Biologically, testosterone in males contributes to greater BMD, whereas estrogen plays a crucial role in maintaining bone density in females (Väänänen and Härkönen, 1996). However, after menopause, a significant drop in estrogen levels can lead to an increased risk of bone loss and osteoporosis in women.

Table 3 summarizes statistics for various physiological and demographic parameters across male, female, and combined datasets. Males display an average ALM of 21.88 kg and a BFP of 25.20%, whereas females show lower ALM (15.77 kg) but higher BFP (34.71%), with the combined data reflecting intermediate values (ALM at 18.68 kg, BFP at 30.19%). Although the distribution of ALM is slightly positively skewed, those of BFP and BMD are balanced across all datasets. The data predominantly consists of younger individuals with a median age of 17, although older individuals raise the mean age to 27.62. The variance for height and weight possess high variance. This variability also indicates diverse physiological measurements and notable individual differences within the sample.

**TABLE 3:** Descriptive statistics for male, female, and combined cohorts, showing mean, median, and standard deviation (SD) for key variables.

	Variable	Mean	Median	SD
Male	ALM (kg)	21.88	23.40	7.95
	BFP (%)	25.20	24.96	7.67
	BMD (g/cm^-2^)	1.07	1.10	0.19
	Age (years)	27.33	17.00	19.59
	Height (cm)	164.33	171.20	17.80
	Weight (kg)	68.24	69.70	25.88
Female	ALM (kg)	15.77	15.50	4.24
	BFP (%)	34.71	34.90	7.37
	BMD (g/cm^-2^)	1.00	1.01	0.14
	Age (years)	27.88	17.00	20.25
	Height (cm)	156.68	158.75	12.47
	Weight (kg)	60.00	58.85	18.22
Combined	ALM (kg)	18.68	17.90	6.98
	BFP (%)	30.19	30.14	8.89
	BMD (g/cm^-2^)	1.03	1.04	0.17
	Age (years)	27.62	17.00	19.92
	Height (cm)	160.32	162.40	15.70
	Weight (kg)	63.92	63.20	22.55

### 4.3 Predictor correlation analysis

Before the usage of data in this machine learning problem, we wanted to make sure that the data did not have correlated predictors solely due to pre-processing or machine artifacts of the measurements taken from the patients. To test this, we performed predictor-predictor correlation analysis using Pearson Correlation Coefficients for all the three data sets, namely Male, Female, and Combined. The correlations analysis revealed that the body parts that are physically related such as the size, area, and volume of one’s arms or legs, exhibit similar trends and were found to be highly correlated. This is not a mistake in the data; it’s simply a natural aspect of human anatomy. Because these patterns remained the same whether we examined Male, Female, and Combined data, we know our data is stable (Dormann et al., 2013). The bilaterally clustered features for each Male, Female and Combined data set are represented as heatmaps in Supplementary Figures S1–S3 respectively. The lists of the most correlated biomarkers for ALM, BFP and BMD are in Tables 4–6.

**TABLE 4:** Top 10 most correlated biomarkers with ALM and their correlation coefficients.

Male	Corr. Coeff	Female	Corr. Coeff	Combined	Corr. Coeff
Surface area total	0.964	Surface area total	0.907	Surface area total	0.910
Surface area leg right	0.956	Leg volume left	0.899	Surface area arm left	0.904
Surface area leg left	0.953	Surface area leg right	0.898	Surface area arm right	0.902
Surface area arm left	0.947	Surface area leg left	0.897	Weight	0.888
Leg volume left	0.944	Leg volume right	0.894	Surface area torso	0.875
Leg volume right	0.943	Weight	0.891	Forearm circumference right	0.872
Surface area arm right	0.939	Volume	0.868	Surface area leg right	0.869
Weight	0.934	Calf circumference left	0.865	Volume	0.865
Volume	0.922	Thigh circumference left	0.862	Surface area leg left	0.865
Surface area torso	0.916	Thigh circumference right	0.861	Arm volume left	0.861

**TABLE 5 T5:** Top 10 most correlated biomarkers with BFP and their correlation coefficients.

Male	Corr. Coeff	Female	Corr. Coeff	Combined	Corr. Coeff
Horizontal waist	0.577	Horizontal waist	0.754	Waist circumference	0.577
Narrow waist	0.552	Waist circumference	0.742	Horizontal waist	0.566
Waist circumference	0.526	Narrow waist	0.741	Abdomen circumference	0.559
Abdomen circumference	0.512	Abdomen circumference	0.741	Seat circumference	0.510
Seat circumference	0.444	Bicep circumference left	0.720	Hip circumference	0.505
Hip circumference	0.442	Chest	0.697	Thigh circumference left	0.479
Bicep circumference left	0.438	Upper arm circumference right	0.691	Thigh circumference right	0.475
Torso volume	0.436	Upper arm circumference left	0.690	MidThigh circumference left	0.459
Chest	0.416	Bicep circumference right	0.688	MidThigh circumference right	0.448
Upper arm circumference left	0.409	Torso volume	0.665	Narrow waist	0.442

**TABLE 6:** Top 10 most correlated biomarkers with BMD and their correlation coefficients.

Male	Corr. Coeff	Female	Corr. Coeff	Combined	Corr. Coeff
Surface area arm right	0.862	Height	0.733	Surface area total	0.806
Surface area arm left	0.861	Surface area total	0.711	Height	0.804
Surface area total	0.851	Surface area leg left	0.709	Surface area arm left	0.795
Arm volume right	0.834	Surface area leg right	0.696	Surface area arm right	0.794
Height	0.834	Outside leg length left	0.675	Surface area leg left	0.782
Forearm circumference right	0.832	Outside leg length right	0.662	Surface area leg right	0.773
Arm volume left	0.831	Arm length left	0.659	Surface area torso	0.767
Surface area leg left	0.829	Surface area torso	0.658	Arm volume right	0.765
Surface area leg right	0.821	Surface area arm left	0.654	Arm volume left	0.758
Surface area torso	0.816	Hip circumference	0.652	Arm length left	0.749

## 5 Supervised learning algorithms

In this section, we present an overview of the supervised algorithms we implemented. For all the models, we use a single, consistent 5-fold cross-validation protocol. In each fold, 80% of the data will be used for training and 20% for testing. Also, to ensure reproducibility and avoid bias, a fixed random seed was used for data splitting and model initialization across all models. In this section, we briefly describe the supervised models we use. We discuss our results for these models in the Results and Analysis section.

We consider Linear (LR) and Polynomial Regression (PR), alongside three of their variations: Lasso, Ridge, and Bayesian. Thevaraja et al. (2019) we also consider Support Vector Regression (SVR) (Awad and Khanna, 2015), and its variant Least Squared SVR (LSSVR) (Suykens and Vandewalle, 1999). Moreover, we study Random Forest (RF) (Genuer and Poggi, 2020), and the Extreme Gradient Boosting (XGBoost) models (Bentéjac et al., 2021). A Multilayer Perceptron is also analyzed, and we chose a ReLU activation function with an Adam optimizer, as these yielded the best performance.

## 6 Semi-supervised learning algorithms

Semi-supervised learning is a category of machine learning algorithms that combines the utility of unsupervised learning and supervised learning algorithms. Supervised algorithms construct a classifier or a regressor by training the dataset to find associations between the input values and the target. Such algorithms then leverage these associations to predict the output value for previously unseen inputs, James et al. (2023). In an unsupervised algorithm, inputs are assigned to clusters based on feature similarity, ensuring that inputs with similar characteristics are grouped. In a semi-supervised algorithm, the relationship between labeled (training) and unlabeled (testing) data is utilized to improve the learning process (James et al., 2023). A semi-supervised algorithm is useful when extracting labeled data is either computationally or financially expensive, as is the case with DXA scans and DXA equipment, which are both costly. Semi-supervised algorithms have traditionally been applied to classification tasks, as their design is often tailored specifically for classification and does not readily extend to regression problems. The class of semi-supervised learning methods known as graph-based methods can be extended to regression problems (Van Engelen and Hoos, 2019). Zhu (2005) is one of the first researchers to introduce graph-based semi-supervised algorithms in their thesis. We implemented a version of a graph-based method using p -Laplacian regression introduced in Calder and Drenska (2024). In this section, we will elucidate the underlying concepts of their model. For the sake of simplicity, we will explain the model for predicting ALM. The same procedure works for BFP and BMD. In the following discussion, by labeled vertex, we refer to a patient whose ALM value the algorithm remembers. Such vertices are in the training set for the algorithm.

We discuss this graph-based method from Calder and Drenska (2024) in two steps: (1) Graph Construction and (2) The Tug-of-War equation.

### 6.1 Graph construction

We follow the standard notation and terminology for the graph. Let G be our graph with n vertices where n is the total number of patients in the dataset. Let $X=\left\{x_{1},x_{2},\ldots ,x_{n}\right\}$ be the set of vectors in $R^{44}$ . Each $x_{i}$ represents a vertex in G . Each entry in $x_{i}$ is a measurement of one of the biomarkers of the patient the vertex represents. The $x_{i}$ ’s are first normalized using a z -score normalizer. We connect two vertices $x_{i}$ and $x_{j}$ with an edge only when they are within the threshold of similarity we define below. For a pair of vertices $x_{i}$ and $x_{j}$ , we define the Euclidean distance, $d\left(x_{i},x_{j}\right)=\|x_{i}-x_{j}\|$ in $R^{44}$ . Intuitively, for the pair of vertices $x_{i}$ and $x_{j}$ , the greater the value of $d\left(x_{i},x_{j}\right)$ is, the less similar the patients corresponding to $x_{i}$ and $x_{j}$ are. Therefore, we need a nonnegative decreasing function η to define the edge weight, $w_{x_{i}x_{j}}$ .

$$w_{x_{i}x_{j}}=\eta \left(\frac{d\left(x_{i},x_{j}\right)}{\epsilon }\right)$$ (2)

where ϵ>0 is a free parameter.

Equation 2 implies $w_{x_{i}x_{j}}=w_{x_{j}x_{i}}$ . Therefore, G is an undirected graph. As used in Calder (2022), we chose $\eta \left(t\right)=e^{-t^{2}}$ as our nonnegative decreasing function. Furthermore, this choice ensures the values of $w_{x_{i}x_{j}}$ lie strictly between 0 and 1. For computational efficiency, we construct a k -nearest neighbors ( k -NN) graph, where each vertex has k neighbors. We follow the method described by Calder and García Trillos (2022) to construct a k -NN graph. We denote the open neighborhood of a vertex $x_{i}$ as $N_{x_{i}}$ . Theoretically, this algorithm of constructing a k -NN works even when G is not connected, provided that each connected component of G has at least one labeled vertex. However, for the computer simulations to run, G must be connected. The primary hypothesis underlying the construction of a similarity-based graph is that individuals with similar measurements of their biomarkers have their ALM within a small margin. We estimate the ALM value of a vertex $x_{i}$ by using the ALM values of the rest of the vertices in G . Vertices in the neighborhood of $x_{i}$ in G have a higher influence on its ALM. The influence of the vertices declines as we move farther from $x_{i}$ in G .

### 6.2 Tug-of-war equation

Like supervised algorithms, p -Laplacian-based regression needs a training set and a testing set. We divide the vertex set, X into two subsets: Γ and X\Γ . For vertices in Γ , we remember the values of the ALM of the corresponding patients, which makes Γ our training set. Contrastingly, for nodes in X\Γ , we temporarily forget the values of the corresponding ALM which makes the set X\Γ our testing set. Let g:Γ→R be the true value of ALM for vertices in Γ and u:X→R be our estimated value of ALM for vertices in X . Clearly, u and g are identical in Γ .

To estimate the value of ALM for the vertices in X\Γ , we solve the Game-Theoretic p -Laplacian for the function u on the set X\Γ . Equation 3 describes the system, which we will solve for *g(x)* for *x* in X\Γ . Hence, the following set of equations describes our system:

$$\left\{\begin{array}{cc} L_{p}u\left(x_{i}\right)=0 & \text{if }x_{i}\in X\backslash \Gamma \\ u\left(x_{i}\right)=g\left(x_{i}\right) & \text{if }x_{i}\in \Gamma . \end{array}\right.$$ (3)

The parameter p in (3) lies in the interval $\left.\left[2,\infty \right.\right)$ . To solve, $L_{p}u\left(x_{i}\right)=0$ , we utilize the Dynamic Programming Principle (DPP) described in Calder and Drenska (2024). DPP can be represented by the following equation:

$$u\left(x_{i}\right)=\frac{\alpha }{d_{x_{i}}}\underset{x_{j}\in N_{x_{i}}}{\sum }w_{x_{i}x_{j}}u\left(x_{j}\right)+\frac{1-\alpha }{2}\left(\underset{N_{x_{i}}}{\min}u+\underset{N_{x_{i}}}{\max}u\right).$$ (4)

Here $\alpha =\frac{1}{p-1}$ and $d_{x_{i}}=\sum_{x_{j}}w_{x_{i}x_{j}}$ denotes the degree of vertex $x_{i}$ . Equation 4 arises from a stochastic two-player tug-of-war game. Further details on this procedure can be found in Calder and Drenska (2024).

Equation 4 shows that when p=2 , we have α=1 , which results in the elimination of the terms associated with the minimum and maximum values of u . Consequently, the model utilizes exclusively the random walk. For higher values of p , the contribution of the first term diminishes. Moreover, as p becomes larger, the factor $\frac{1-\alpha }{2}$ approaches $\frac{1}{2}$ .

We use the GraphLearning Python package Calder (2022) to perform semi-supervised p -Laplacian regression for predicting ALM from the 44 biomarker measurements stored in $X_{\text{combined}}\in R^{n\times 44}$ . The method first builds a weighted k -nearest neighbor ( k -NN) graph on the patient set: for each vertex (patient) i , edges connect i to its k nearest neighbors in biomarker space and the corresponding affinities are stored in a weight matrix W . Given a labeled (training) index set L and observed ALM values ${\left\{y_{i}\right\}}_{i\in L}$ , we then solve the p -Laplacian regression problem on this graph to obtain predictions $\hat{y}$ for all vertices. By construction, the solution satisfies ${\hat{y}}_{i}=y_{i}$ for every labeled vertex i∈L .

Algorithm 1 *p*-Laplacian Regression for ALM Prediction with GraphLearning.

**Require:** Biomarker matrix $X_{\text{combined}}\in R^{n\times 44}$ ; ALM vector $y_{\text{combined}}\in R^{n}$ ; labeled/training index set L⊆{1,…,n} ; neighborhood size k ; exponent p

**Ensure:** Predictions $\hat{y}\in R^{n}$

 1: **Weight matrix and graph construction**
 2: $W\leftarrow gl.weightmatrix.knn\left(X_{\text{combined}},\;k\right)$

 3: G←gl.graph(W)

 4: p -**Laplacian regression (semi-supervised)**
 5: $\hat{y}\leftarrow G.plaplace\left(L,\;y_{\text{combined}}\left[L\right],\;p\right)$

 6: **Label consistency on training vertices**
 7: ⊳ ${\hat{y}}_{i}=y_{i}$ for all i∈L by construction
 8: **return** $\hat{y}$

In Section 7.2, we discuss our results on p -Laplacian based regression.

## 7 Results and analysis

In this section, we present our results for both supervised and semi-supervised algorithms. Our goal is to develop predictive regression models for estimating ALM, BMD, and BFP from limited labeled data. Hence, we use RMSE as the primary metric to assess relative predictive accuracy across models and cross-validation folds.

### 7.1 Supervised algorithms

We implemented the supervised algorithms with K -cross validation with K=5 . Tables 7–9 include the optimal parameters of the supervised algorithms with their respective errors for ALM, BMD, and BFP respectively.

**TABLE 7:** Optimal parameters and corresponding RMSEs of supervised algorithms for ALM.

Supervised algorithms (ALM)				
Model	Par./RMSE	Male	Female	Combined
Regression	Par. RMSE	Traditional 8.00	Ridge 9.10	Bayesian 9.00
LSSVR	Par	γ : 0.001, C : 500	γ : 0.001, C : 1000	γ : 0.001, C : 1000
	RMSE	7.47	6.03	8.46
NN	Par	Epochs: 150	Epochs: 350	Epochs: 250
	RMSE	14.98	14.01	15.05
RF	Par	n : 50, d : 15	n : 50, d : 15	n : 45, d : 25
	RMSE	7.96	10.52	10.06
SVR	Par	ϵ : 0.7, C : 50	ϵ : 0.3, C : 1.0	ϵ : 0.4, C : 10
	RMSE	6.30	8.97	7.83
XGBoost	Par	n : 35, d : 05	n : 35, d : 05	n : 35, d : 05
	RMSE	8.36	10.82	10.27

**TABLE 8:** Optimal parameters and corresponding RMSEs of supervised algorithms for BFP.

Supervised algorithms (BFP)				
Model	Par./RMSE	Male	Female	Combined
Regression	Par. MSE	Ridge 18.00	Bayesian 14.60	Bayesian 17.00
LSSVR	Par	γ : 0.01, C : 25	γ : 0.01, C : 25	γ : 0.001, C : 250
	RMSE	11.47	12.09	10.99
NN	Par	Epochs: 150	Epochs: 150	Epochs: 350
	RMSE	17.70	13.59	15.75
RF	Par	n : 50, d : 15	n : 50, d : 20	n : 50, d : 15
	RMSE	15.92	12.44	14.13
SVR	Par	ϵ : 0.5, C :0.7	ϵ : 1.0, C : 0.1	ϵ : 1.0, C : 1.0
	RMSE	14.92	11.72	13.28
XGBoost	Par	n : 35, d : 05	n : 30, d : 05	n : 35, d : 05
	RMSE	16.40	12.89	14.55

**TABLE 9:** Optimal Parameters and Corresponding parameters of supervised algorithms for BMD.

Supervised algorithms (BMD)				
Model	Par./RMSE	Male	Female	Combined
Regression	Par RMSE	Ridge 8.10	Bayesian 9.25	Ridge 8.00
LSSVR	Par	γ : 0.001, C : 250	γ : 0.001, C : 100	γ : 0.01, C : 10
	RMSE	7.59	7.05	8.09
NN	Par	Epochs: 250	Epochs: 400	Epochs: 250
	RMSE	10.04	11.04	10.71
RF	Par	n : 45, d : 15	n : 50, d : 20	n : 50, d : 10
	RMSE	7.72	8.09	7.94
SVR	Par	ϵ : 0.05, C : 0.2	ϵ : 0.05, C : 0.05	ϵ : 0.05, C : 0.4
	RMSE	6.97	8.36	7.48
XGBoost	Par	n : 20, d : 05	n : 15, d : 05	n : 20, d : 05
	RMSE	8.22	8.37	8.31

### 7.2 p-Lapacian based regression

We tested the p -Laplacian based regression method under two schemes and training-testing ratios. As described in Section 6, the model is implemented on a graph where the vertices are the patients and the edges are constructed based on the similarity between the patients. We constructed the graph using two methods. In the first method, we used all 44 biomarkers to define similarity and then construct the graph. In the second method, we used the ten most correlated biomarkers with each target variable and dataset listed in Tables 4–6. The idea behind this is to see if using fewer and more significant biomarkers improves the model’s performance. We call the first method p -Laplacian-1 and the second method p -Laplacian-2. Like the supervised models, p -Laplacian-1 and p -Laplacian-2 are implemented with K -fold cross validation with K=5 .

To test the model’s performance when trained on a smaller percentage of training data, we modified the K -fold cross-validation slightly. We used one fold as our training set and the other four as our testing set. We tested both versions of the model using the modified K -fold cross-validation for K=2,3,4,5,10 , and 20 with training testing ratios 50-50, 33-67,25-75, 20-80, 10-90, and 5-95, respectively.

**FIGURE 2:**
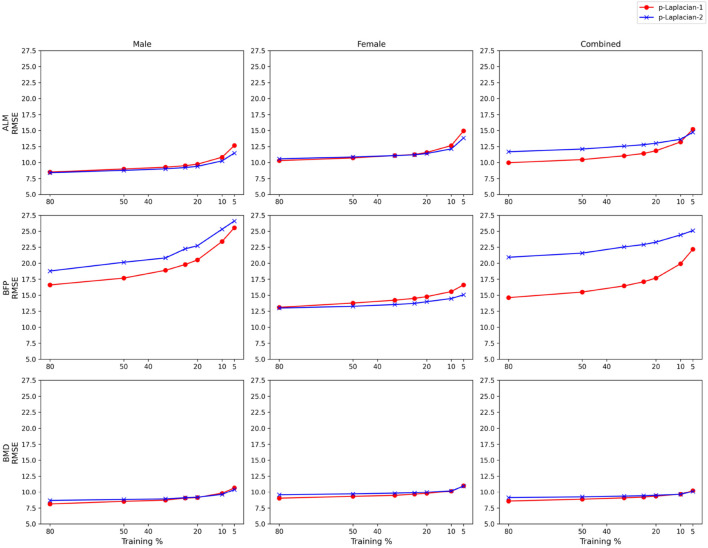
RMSEs (in %) vs. Training % of p -Laplacian-1 and p -Laplacian-2 across the target variables and datasets.

In the following discussion, we represent the percentage of training data as Training %. We vary two parameters: p (see the description of Equation 4) and k (the number of neighbors every vertex has in the graph). For optimization, we implemented p -Laplacian-1 and p -Laplacian-2 for different combinations of p and k for a fixed Training %. For both models, the optimal p is at most 10 and the optimal k is at most 60. Figure 2 demonstrates how the lowest RMSE p -Laplacian-1 and p -Laplacian-2 yielded changes with Training %. The RMSEs of both models increase as the Training % decreases. This phenomenon is not unusual. Calder and Drenska (2024), showed for a random graph like k -NN graph the accuracy of the p -Laplacian-based algorithm can decline with label rate which is equivalent to Training %. There is a relatively steep increase in the models’ RMSE when the Training % is reduced from 10 to 5 across all target variables and datasets. Among the target variables, they both perform the best for BMD. The yielded RMSE is less than 10.2% for all datasets when the Training % is greater than or equal to 10. The RMSEs of both models follow similar trajectories for BMD. For Male ALM and Female ALM, p -Laplacian-2 does slightly better than p -Laplacian-1; however, for Combined ALM, it yields higher RMSEs than p -Laplacian-1 when Training % is at least 10. Like the supervised models, p -Laplacian-1 and p -Laplacian-2 perform poorly on estimating BFP, especially for Male and Combined. p -Laplacian-1 outperforms p -Laplacian-2 by a huge margin when estimating BFP for these two datasets. Both p -Laplacian-1 and p -Laplacian-2 perform well for BMD yielding errors below 10% for all the datasets even when they are trained on only 10 percent of the data. The errors are slightly above 10% when Training % is reduced to 5%.

**FIGURE 3:**
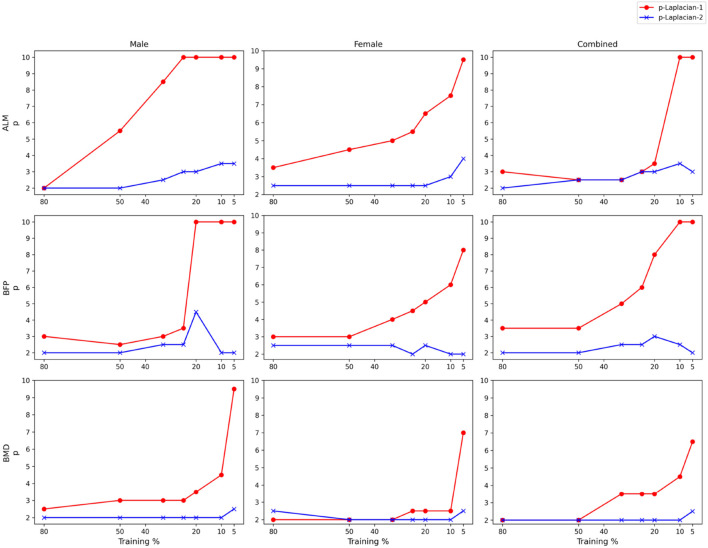
Optimal p vs. Training % of p -Laplacian-1 and p -Laplacian-2 across target variables and datasets.

**FIGURE 4:**
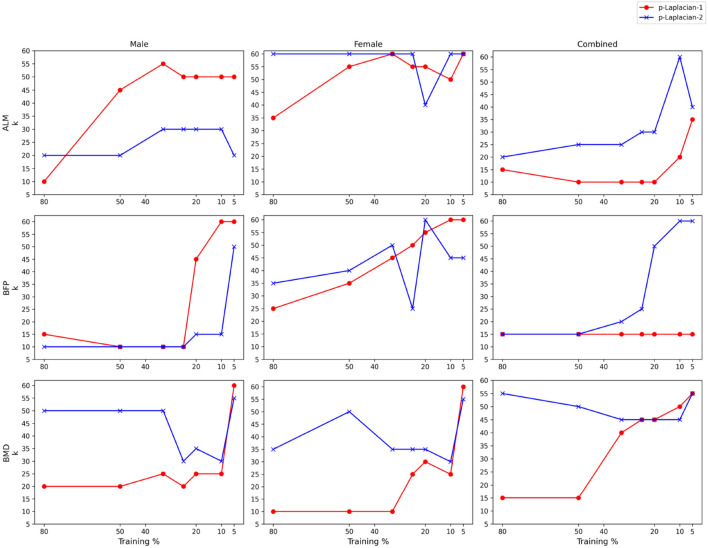
Optimal k vs. Training % of p -Laplacian-1 and p -Laplacian-2 across target variables and datasets.

Figures 3, 4 demonstrate how the optimal parameters p and k change for p -Laplacian-1 and p -Laplacian-2, respectively with the Training %. As illustrated by Figure 3, when Training % is 10 or 5, the optimal p values for p -Laplacian-1 and p -Laplacian-2 jump above 2 for ALM and BMD. In the case of BFP, this happens for p -Laplacian-1 but not for p -Laplacian-2. Calder et al. (2023) proved that at low label rates (equivalent to Training %), a 2-Laplacian algorithm (p=2) becomes degenerate which implies that it fails to predict the target variables with high accuracy. Another observation is that p -Laplacian-1 is optimized at much higher p values when compared to p -Laplacian-2. We did not find any evidence on why p -Laplacian-2 acts this way for BFP. One reason could be that weakly correlated biomarkers with the target variables make the convergence of the RMSEs slower. Further investigation is required to substantiate this hypothesis.

Figure 4 demonstrates the optimal k values for both p -Laplacian models. There is no obvious pattern to distinguish between the optimal k values of p -Laplacian-1 and p -Laplacian-2.

**FIGURE 5:**
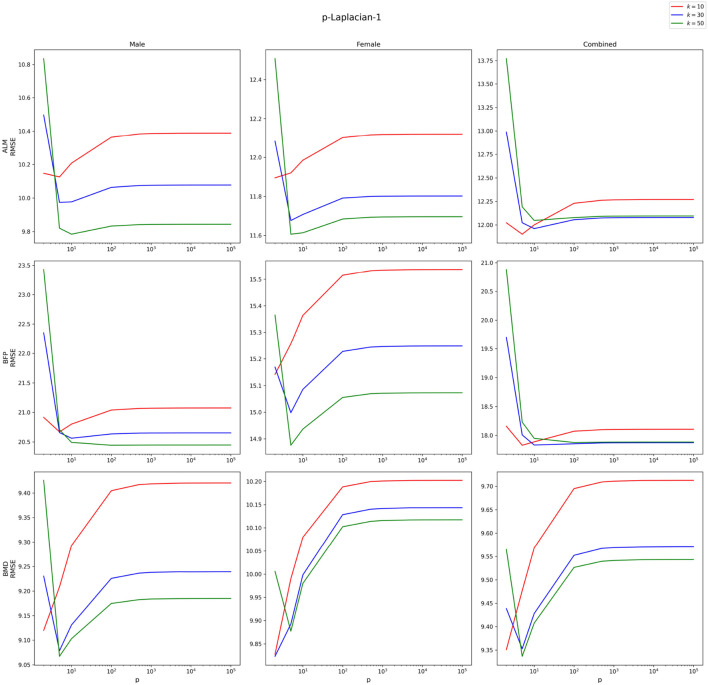
Asymptotic behavior of p -Laplacian-1 as p→∞ for k=10,30 ,and 50.

**FIGURE 6:**
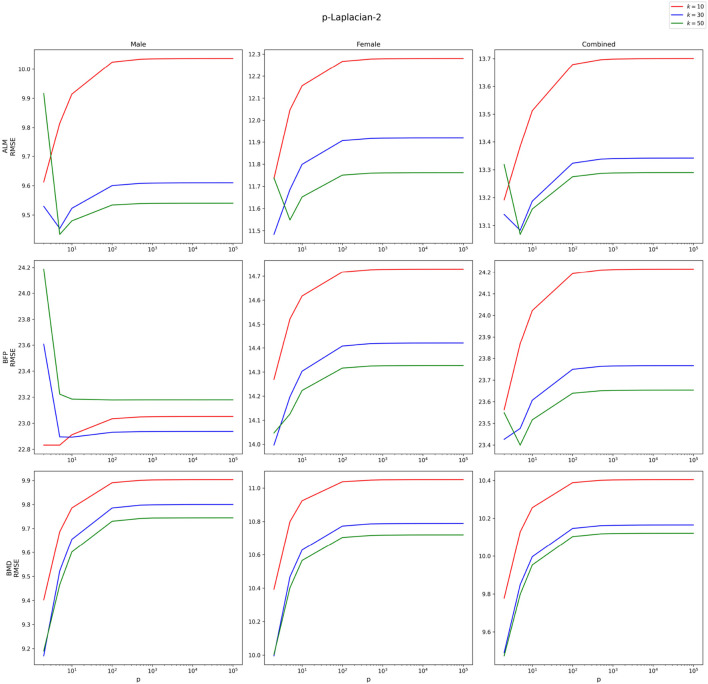
Asymptotic behavior of p -Laplacian-2 as p→∞ for k=10,30 , and 50.

Since p can approach infinity, it is natural to investigate the asymptotic behavior of p -Laplacian based regression. We implemented p -Laplacian-1 and p -Laplacian-2 for larger values of p by fixing k=10,30 , and 50. Figures 5, 6 demonstrate the asymptotic behavior of RMSEs of p -Laplacian-1 and p -Laplacian-2 respectively when p approaches infinity. For demonstration, Training % is fixed at 20%; however, similar results are achieved for other values of Training %. As Figures 5, 6 demonstrate, the RMSEs converge as p gets larger.

The detailed summary on the performances of p -Laplacian-1 and p -Laplacian-2 can be found in Supplementary Tables S4–S10.

## 8 Discussion

This article has two main objectives. The first is to test if ALM, BFP, and BMD can be predicted by applying supervised and semi-supervised algorithms on biomarkers’ obtained by the procedure described in Section 3.1. We used six different supervised algorithms, namely-Linear/Polynomial Regression, SVR, LSSVR, Random Forest, XGBoost and Neural Network. The second objective is to determine whether accurate prediction of these biomarkers is possible with limited labeled data. To achieve this, we used a p -Laplacian based regression, a semi-supervised algorithm.

The supervised models demonstrated significantly higher accuracy for ALM and BMD compared to BFP. The BFP predictions for Female were on average better than those for Male and Combined. One of the reasons behind models’ poor performance on predicting BFP could be its relatively low correlations with the biomarkers as indicated in Table 5. The SVR and LSSVR models are the most accurate models for all target variables across all datasets. Table 10 summarizes the best performing supervised models with their respective errors.

**TABLE 10:** Summary of the best performing Supervised Models for the Target variables with respective RMSEs (in %).

	ALM		BFP		BMD	
	Model	RMSE	Model	RMSE	Model	RMSE
Male	SVR	6.30	LSSVR	11.47	SVR	6.97
Female	LSSVR	6.03	SVR	11.72	LSSVR	7.05
Combined	SVR	7.83	LSSVR	10.99	SVR	7.48

Our results show that SVR and LSSVR have the potential to be alternatives to DXA scans for estimating ALM and BMD.

As we have discussed earlier, obtaining body composition data using a DXA scanner is time-consuming, requiring specialized equipment and prolonged acquisition procedures. In contrast, once we have extracted biomarkers from 3D avatar representations, the computational cost of training the proposed models on 3D-derived biomarkers is relatively low (see Table A1), further highlighting the efficiency of the proposed pipeline.

In contrast to Feng et al. (2026), which evaluates performance for using a single train-validation split without a separate test set, our framework incorporates an explicit test set, containing both adults and children, and utilizes repeated K -fold cross-validation with varying training-testing ratios for the p -Laplacian model. Additionally, we report normalized RMSE (in %), instead of unnormalized mean absolute error (mean absolute error is always smaller than or equal to RMSE) enabling scale-invariant comparison across targets of different magnitudes.

Compared to the supervised regression algorithms we implemented in this paper, p -Laplacian based regression is not so well studied. In our analysis, p -Laplacian demonstrates that in the future, researchers could leverage it effectively, especially when obtaining training data is computationally or financially expensive. Like the supervised models, both p -Laplacian-1 and p -Laplacian-2 perform considerably better for ALM and BMD compared to BFP. The errors are below 10% even when the training percent is significantly low. To predict BFP more accurately using p -Laplacian based regression, a higher correlated set of biomarkers with BFP could be used to construct the graph.

It is compelling to examine how the performance of the p -Laplacian model changes with the size of the dataset for a fixed proportion of training data. This way we can test if the number of data points in training impacts the model’s performance differently than the proportion of training data.

Furthermore, in our analysis, all the biomarkers are weighed equally to construct the similarity-based graph. Hence, one potential direction for future research is weighing the biomarkers differently. One such weighing could be based on how correlated they are with the target variable.

In summary, our results show DXA could be potentially replaced in the future to estimate ALM and BMD. However, further investigation is needed on larger datasets. Additionally, our investigation of the p -Laplacian model yielded positive results for ALM and BMD in terms of reducing the amount of training data needed while maintaining a high level of accuracy. Our analysis opens new avenues to implement and analyze our p -Laplacian-based model in regression problems.

### 8.1 Limitations

This study has several important limitations. A key one concerns the *size and representativeness* of the analytic cohort. Although 847 participants’ data were initially collected, the final modeling analyses were performed on 515 individuals after removing entries with missing values. This listwise-deletion approach reduces statistical power and may introduce selection bias if missingness is not completely at random. In addition, data were collected at a single research center, and the age distribution is skewed relatively young (median 17, with a higher mean driven by older participants), which limits external generalization to broader or older clinical populations without independent validation on external cohorts.

We examine sex-stratified performance throughout the paper–every method that was tested was implemented for males, females, and a combined data set. Race and ethnic differences in body composition are very small and can only be detected with large samples, Heymsfield et al. (2016). Moreover, self-reported race and ethnicity are fluid concepts as they are impacted by cultural and genetic factors. Many people in Louisiana, where the data was collected, have mixed genetic backgrounds.

Methodologically, the semi-supervised p -Laplacian framework introduces *graph- and hyperparameter-sensitivity* that constrain interpretation and deployment. In particular, the k -NN similarity graph must be connected for the simulations, and performance depends on the choice of k and p , with error increasing as the labeled fraction decreases and sharp degradation between 10% and 5% labeled data. Moreover, prediction quality varies by outcome: BFP is consistently harder to predict, in accordance with with weaker correlations between BFP and the available biomarkers, and some observed behaviors of the p -Laplacian variants for BFP remain unexplained. Finally, similarity construction currently weights all biomarkers equally, suggesting that more principled feature weighting and larger multi-site datasets are important next steps to strengthen robustness and reproducibility.

## Data Availability

The raw data supporting the conclusions of this article will be made available by the authors, without undue reservation.

## Supplementary material

SUPPLEMENTARY FIGURE S1 Hierarchical clustering heatmap of predictor–predictor correlations computed using male subjects only. Similar correlation blocks are observed across related body measurements, confirming that multicollinearity patterns are stable across male subsets and arise from physiological redundancy.

SUPPLEMENTARY FIGURE S2 Hierarchical clustering heatmap of predictor–predictor correlations computed using female subjects only.

SUPPLEMENTARY FIGURE S3 Hierarchical clustering heatmap of pairwise correlations among all anthropometric predictors for the complete dataset. Strong block-wise correlation patterns reflect anatomical and biomechanical groupings, indicating structured multicollinearity rather than feature leakage.

## Appendix

**TABLE A1:** CPU and wall-clock computation time for each model. CPU time is training time reported across three datasets, while wall-clock time corresponds to the total elapsed training and testing time.

Model	CPU time (seconds)			Wall-clock time (seconds)
	Male	Female	Combined	
Linear/Polynomial	0.0805	0.0601	0.1038	0.7
SVR	4.565	0.281	2.918	110
LSSVR	0.084	0.091	3.689	52.2
XGBoost	1377.553	657.083	25.523	4195.2
RF	7.80	8.15	11.15	9747.4
Neural network	32.861	55.772	99.621	16432.8
p-Laplacian	62.72	67.06	113.43	190.95
