# Supplementary material for: Predicting anthropometric body composition variables using 3D optical imaging and machine learning
Source: Front Bioinform. 2026 May 13;6:1722578. doi: 10.3389/fbinf.2026.1722578 (PMC13213389; doi:10.3389/fbinf.2026.1722578)
Supplement: Supplementary file 1 [file Supplementaryfile1.pdf]

Supplementary Material

1 SUPPLEMENTARY TABLES

Tables S4, S5, and S6 include the list of the optimal parameters p and k for p-Laplacian-1 model with their respective errors. Tables S7, S8, and S9 do the same for p-Laplacian-2 model.

Table S1. Optimal Parameters and Corresponding RMSEs of Supervised Algorithms for ALM

Supervised Algorithms (ALM)				
**Model**	**Par./RMSE**	**Male**	**Female**	**Combined**
Regression	Par.<br>RMSE	Traditional<br>8.00	Ridge<br>9.10	Bayesian<br>9.00
LSSVR	Par.<br>RMSE	γ: 0.001, C: 500<br>7.47	γ: 0.001, C: 1000<br>6.03	γ: 0.001, C: 1000<br>8.46
NN	Par.<br>RMSE	Epochs: 300<br>7.20	Epochs: 300<br>9.85	Epochs: 200<br>8.70
RF	Par.<br>RMSE	n: 50, d: 15<br>7.96	n: 50, d: 15<br>10.52	n: 45, d: 25<br>10.06
SVR	Par.<br>RMSE	ϵ: 0.7, C: 50<br>6.30	ϵ: 0.3, C: 1.0<br>8.97	ϵ: 0.4, C: 10<br>7.83
XGBoost	Par.<br>RMSE	n: 35, d: 05<br>8.36	n: 35, d: 05<br>10.82	n: 35, d: 05<br>10.27

Table S2. Optimal Parameters and Corresponding RMSEs of Supervised Algorithms for BFP

Supervised Algorithms (BFP)				
**Model**	**Par./RMSE**	**Male**	**Female**	**Combined**
Regression	Par.<br>RMSE	Ridge<br>18.00	Bayesian<br>14.60	Bayesian<br>17.00
LSSVR	Par.<br>RMSE	γ: 0.01, C: 25<br>11.47	γ: 0.01, C: 25<br>12.09	γ: 0.001, C: 250<br>10.99
NN	Par.<br>RMSE	Epochs: 300<br>14.20	Epochs: 400<br>12.00	Epochs: 250<br>12.80
RF	Par.<br>RMSE	n: 50, d: 15<br>15.92	n: 50, d: 20<br>12.44	n: 50, d: 15<br>14.13
SVR	Par.<br>RMSE	ϵ: 0.5, C:0.7<br>14.92	ϵ: 1.0, C: 0.1<br>11.72	ϵ: 1.0, C: 1.0<br>13.28
XGBoost	Par.<br>RMSE	n: 35, d: 05<br>16.40	n: 30, d: 05<br>12.89	n: 35, d: 05<br>14.55

Table S3. Optimal Parameters and Corresponding parameters of supervised algorithms for BMD

Supervised Algorithms (BMD)				
**Model**	**Par./RMSE**	**Male**	**Female**	**Combined**
Regression	Par.<br>RMSE	Ridge<br>8.10	Bayesian<br>9.25	Ridge<br>8.00
LSSVR	Par.<br>RMSE	γ: 0.001, C: 250<br>7.59	γ: 0.001, C: 100<br>7.05	γ: 0.01, C: 10<br>8.09
NN	Par.<br>RMSE	Epochs: 200<br>7.35	Epochs: 350<br>8.60	Epochs: 400<br>7.90
RF	Par.<br>RMSE	n: 45, d: 15<br>7.72	n: 50, d: 20<br>8.09	n: 50, d: 10<br>7.94
SVR	Par.<br>RMSE	ϵ: 0.05, C: 0.2<br>6.97	ϵ: 0.05, C: 0.05<br>8.36	ϵ: 0.05, C: 0.4<br>7.48
XGBoost	Par.<br>RMSE	n: 20, d: 05<br>8.22	n: 15, d: 05<br>8.37	n: 20, d: 05<br>8.31

Table S4. Optimal Parameters and Corresponding RMSEs of p-Laplacian-1 for ALM

**p-Laplacian-1 (ALM)**								
**Dataset**	**Par./RMSE**	**80%**	**50%**	**33%**	**25%**	**20%**	**10%**	**5%**
Male	p	2.0	5.5	8.5	10.0	10.0	10.0	10.0
	k	10	45	55	50	50	50	50
	RMSE	8.51	8.99	9.27	9.50	9.74	10.83	12.66
Female	p	3.5	4.5	5.0	5.5	6.5	7.5	9.5
	k	35	55	60	55	55	50	60
	RMSE	10.30	10.72	11.10	11.25	11.57	12.65	14.95
Combined	p	3.0	2.5	2.5	3.0	3.5	10.0	10.0
	k	15	10	10	10	10	20	35
	RMSE	9.97	10.46	11.05	11.42	11.84	13.21	15.20

Table S5. Optimal Parameters and Corresponding RMSEs of p-Laplacian-1 for BFP

**p-Laplacian-1 (BFP)**								
**Dataset**	**Par./RMSE**	**80%**	**50%**	**33%**	**25%**	**20%**	**10%**	**5%**
Male	p	3.0	2.5	3.0	3.5	10.0	10.0	10.0
	k	15	10	10	10	45	60	60
	RMSE	16.61	17.68	18.90	19.80	20.51	23.40	25.56
Female	p	3.0	3.0	4.0	4.5	5.0	6.0	8.0
	k	25	35	45	60	55	60	60
	RMSE	13.11	13.78	14.22	14.51	14.78	15.57	16.62
Combined	p	3.5	3.5	5.0	6.0	8.0	10.0	10.0
	k	15	15	15	15	15	15	15
	RMSE	14.62	15.50	16.46	17.10	17.68	19.92	22.19

Table S6. Optimal Parameters and Corresponding RMSEs of p-Laplacian-1 for BMD

**p-Laplacian-1 (BMD)**								
**Dataset**	**Par./RMSE**	**80%**	**50%**	**33%**	**25%**	**20%**	**10%**	**5%**
Male	p	2.5	3.0	3.0	3.0	3.5	4.5	9.5
	k	20	20	25	20	25	45	60
	RMSE	8.11	8.52	8.71	9.03	9.11	9.81	10.68
Female	p	2.0	2.0	2.0	2.5	2.5	2.5	7.0
	k	10	10	10	25	30	25	60
	RMSE	9.02	9.30	9.46	9.65	9.79	10.13	11.00
Combined	p	2.0	2.0	3.5	3.5	3.5	4.5	6.5
	k	15	15	40	40	45	50	55
	RMSE	8.57	8.86	9.06	9.19	9.34	9.65	10.23

Table S7. Optimal Parameters and Corresponding RMSEs of p-Laplacian-2 for ALM

p-Laplacian-2 (ALM)								
**Dataset**	**Par./RMSE**	**80%**	**50%**	**33%**	**25%**	**20%**	**10%**	**5%**
Male	p	2.0	2.0	2.5	3.0	3.0	3.0	3.5
	k	20	20	30	30	30	30	20
	RMSE	8.41	8.78	9.01	9.22	9.40	10.26	11.48
Female	p	2.5	2.5	2.5	2.5	2.5	3.0	4.0
	k	60	60	60	60	40	60	60
	RMSE	10.57	10.87	11.07	11.20	11.41	12.13	13.83
Combined	p	2.0	2.5	2.5	3.0	3.0	3.5	3.0
	k	20	25	25	30	30	60	40
	RMSE	11.69	12.11	12.56	12.79	13.02	13.64	14.72

Table S8. Optimal Parameters and Corresponding RMSEs of p-Laplacian-2 for BFP

p-Laplacian-2 (BFP)								
**Dataset**	**Par./RMSE**	**80%**	**50%**	**33%**	**25%**	**20%**	**10%**	**5%**
Male	p	2.0	2.0	2.5	2.5	4.5	2.0	2.0
	k	10	10	10	10	15	15	50
	RMSE	18.79	20.15	20.83	22.25	22.74	25.32	26.60
Female	p	2.5	2.5	2.5	2.0	2.5	2.0	2.0
	k	35	40	50	25	60	45	45
	RMSE	12.99	13.27	13.54	13.72	13.99	14.48	15.07
Combined	p	2.0	2.0	2.5	2.5	3.0	2.5	2.0
	k	15	15	20	25	50	60	60
	RMSE	20.94	21.59	22.54	22.92	23.30	24.43	25.10

Table S9. Optimal Parameters and Corresponding RMSEs of p-Laplacian-2 for BMD

p-Laplacian-2 (BMD)								
**Dataset**	**Par./RMSE**	**80%**	**50%**	**33%**	**25%**	**20%**	**10%**	**5%**
Male	p	2.0	2.0	2.0	2.0	2.0	2.0	2.5
	k	50	50	50	30	35	30	55
	RMSE	8.66	8.82	8.90	9.08	9.17	9.61	10.41
Female	p	2.5	2.0	2.0	2.0	2.0	2.0	2.5
	k	35	50	35	35	35	30	55
	RMSE	9.55	9.69	9.81	9.89	9.98	10.19	10.96
Combined	p	2.0	2.0	2.0	2.0	2.0	2.0	2.5
	k	55	50	45	45	45	45	55
	RMSE	9.11	9.22	9.34	9.39	9.47	9.63	10.09
